# The Impact of Three-Dimensional Effects on the Simulation of Turbulence Kinetic Energy in a Major Alpine Valley

**DOI:** 10.1007/s10546-018-0341-y

**Published:** 2018-02-23

**Authors:** Brigitta Goger, Mathias W. Rotach, Alexander Gohm, Oliver Fuhrer, Ivana Stiperski, Albert A. M. Holtslag

**Affiliations:** 1 Department of Atmospheric and Cryospheric Sciences, University of Innsbruck, Innsbruck, Austria; 2 Federal Office for Meteorology and Climatology (MeteoSwiss), Zurich, Switzerland; 3 Meteorology and Air Quality Section, Wageningen University, Wageningen, The Netherlands

**Keywords:** Complex terrain, High-resolution atmospheric modelling, Model evaluation, Turbulence kinetic energy, Turbulence parametrization

## Abstract

The correct simulation of the atmospheric boundary layer (ABL) is crucial for reliable weather forecasts in truly complex terrain. However, common assumptions for model parametrizations are only valid for horizontally homogeneous and flat terrain. Here, we evaluate the turbulence parametrization of the numerical weather prediction model COSMO with a horizontal grid spacing of $\Delta x = 1.1\,\hbox {km}$ for the Inn Valley, Austria. The long-term, high-resolution turbulence measurements of the i-Box measurement sites provide a useful data pool of the ABL structure in the valley and on slopes. We focus on days and nights when ABL processes dominate and a thermally-driven circulation is present. Simulations are performed for case studies with both a one-dimensional turbulence parametrization, which only considers the vertical turbulent exchange, and a hybrid turbulence parametrization, also including horizontal shear production and advection in the budget of turbulence kinetic energy (TKE). We find a general underestimation of TKE by the model with the one-dimensional turbulence parametrization. In the simulations with the hybrid turbulence parametrization, the modelled TKE has a more realistic structure, especially in situations when the TKE production is dominated by shear related to the afternoon up-valley flow, and during nights, when a stable ABL is present. The model performance also improves for stations on the slopes. An estimation of the horizontal shear production from the observation network suggests that three-dimensional effects are a relevant part of TKE production in the valley.

## Introduction

Numerical weather prediction (NWP), together with the rise of computational power, has undergone significant improvements in recent years (Bauer et al. 2015). Nowadays, operational NWP models have horizontal grid spacings on the order of $\Delta x=1\, \hbox {km}$ over larger domains (Ziemiański et al. 2011; Leutwyler et al. 2016). This brings improved representation of small-scale, inhomogeneous terrain, leading to a better representation of the atmospheric boundary layer (ABL) structure in NWP models.

A broad range of ABL processes in mountainous terrain were observed in a steep Alpine valley during MAP-RIVERA (Rotach et al. 2004; Rotach and Zardi 2007), a part of the Mesoscale Alpine Programme (MAP, Bougeault et al. 2001). The findings give insight into radiation (Matzinger et al. 2003), the structure of the thermally-driven circulations (Rotach et al. 2008), and the turbulence kinetic energy (TKE) distribution in the valley (Weigel et al. 2007). Other ABL measurement campaigns explored small-scale processes in a crater basin (METCRAX, Whiteman et al. 2008; Lehner et al. 2015), turbulence and the evening transition over inhomogeneous terrain (BLLAST, Lothon et al. 2014), and the ABL structure near an isolated desert mountain (MATERHORN, Fernando et al. 2015).

Running operational weather forecasts over complex terrain such as the Alps requires small horizontal grid spacing. The operational set-up of the COSMO (COnsortium for Small-Scale MOdelling) model operated by the Swiss Federal Office for Meteorology and Climatology (MeteoSwiss) runs with a horizontal grid spacing of $\Delta x=1.1\,\hbox {km}$ (de Morsier et al. 2012), where many ABL processes (e.g. shallow convection), which need to be parametrized on a coarser grid, are already resolved. On the other hand, a horizontal grid spacing of $\Delta x=1.1 \,\hbox {km}$ lies in the so-called “grey zone” for turbulence representation, which leads to the problem that modelled atmospheric turbulence consists of a resolved and a subgrid part (Wyngaard 2004; Honnert et al. 2011). Nevertheless, Zhou et al. (2014) stressed that grey-zone simulations in complex terrain are essential and should not be omitted, because small horizontal grid spacing lying in this range leads to improved terrain, land-use, and soil representation (Holtslag et al. 2013), thus also improving the ABL simulation. This is in agreement with Chow et al. (2006), who conducted simulations of the ABL structure in the Rivera Valley, and showed that especially the representation of soil moisture with a sufficiently small horizontal grid spacing is crucial for the correct simulation of the thermally-driven circulation. The importance of the underlying terrain is also highlighted by Wagner et al. (2014), who performed idealized simulations with varying horizontal grid spacings over an idealized two-ridge topography and suggest that the correct terrain representation may be even more important than the choice of the ABL scheme.

However, it should be noted that common parametrizations in NWP models may not be suitable for complex terrain, since they were developed based on assumptions that are, strictly speaking, only valid for horizontally homogeneous and flat terrain. For example, most high-resolution NWP models still use TKE closure schemes that only consider the vertical turbulent exchange. However, three-dimensional (3D) effects such as horizontal shear production and advection are relevant source terms of TKE in complex terrain (Arnold et al. 2014). Correspondingly, Muñoz-Esparza et al. (2015) state that one-dimensional (1D) turbulence parametrizations lack relevant horizontal TKE generation mechanisms in dynamically-driven situations (e.g., mountain waves) over mountainous terrain. Similar shortcomings were found for foehn winds, where Zängl et al. (2008) suggest that the 1D ABL parametrization underestimates vertical mixing in narrow valleys. A comparison of aircraft TKE measurements above Salt Lake City, USA, with modelled TKE (with and without horizontal shear production) showed that the TKE is far better represented in the simulation with 3D shear production (Zhong and Chow 2013, their Fig. 10.6). Couvreux et al. (2016) compared TKE observations from the BLLAST campaign with NWP model output to find that the model with the smallest horizontal grid spacing (2.5 km) simulated the detailed diurnal TKE structure adequately, but underestimated near-surface TKE, which is in agreement with the comparison simulated TKE of the COSMO model with observations in the Inn Valley, Austria (Goger et al. 2016). Although it is often noted in many studies that 3D effects may be important for the ABL structure in complex terrain, none of these studies actually investigated the impact of 3D processes on TKE production in greater detail.

In complex terrain, both the valley and slope flows contribute to horizontal exchange mechanisms, as shown in high-resolution idealized simulations (Wagner et al. 2015b; Leukauf et al. 2016). ABL observations from the Rivera Valley suggest that sharp gradients in wind speed relate to the up-valley flow lead to significant (horizontal) shear production mechanisms (Weigel and Rotach 2004). In Stiperski and Calaf (2017), it was found that shear-generated turbulence is more anisotropic than buoyancy-driven turbulence, and is characterized by two-component behaviour. Therefore, the correct parametrization of shear-driven turbulence, such as found in a valley, requires two length scales. The length scale associated with the horizontal shear production within the up-valley flow is dependent on the valley width.

This of course raises the question as to what degree the models are able to correctly represent the processes taken into account in the TKE budget equation. The budget terms governing TKE evolution, such as buoyancy production/consumption, vertical turbulent transport, dissipation, and shear production, are seldom observed, and are rarely evaluated in detail—and if, then only during a limited period of time (e.g., Nadeau et al. 2013; Nilsson et al. 2016). Typical model performance studies with a focus on the ABL mostly evaluate classic atmospheric parameters such as mean wind speed, temperature, and various vertical profiles determined from remote sensing systems (Gohm et al. 2004; Schmidli et al. 2009; Kalverla et al. 2016). The evaluation of the TKE closure of a model is not an easy task, because it is necessary to validate also the modelled radiation, turbulent fluxes, and TKE, i.e. the driving fields for the mean ABL structure.

In the present study, we explore the representation of TKE in a high-resolution NWP model and compare it with long-term, high resolution turbulence measurements from representative sites in mountainous terrain (e.g., valley floor, north- and south-facing slopes). The observations also include the TKE budget terms. Therefore, we are able to perform a detailed evaluation of the standard 1D turbulence parametrization of the COSMO model in complex terrain and suggest possible improvements by considering a parametrization including 3D effects.

## Data and Methods

### The Inn Valley

Our location of interest is the Inn Valley in Austria, a mostly west–east oriented major valley in the eastern Alps. The Inn River flows through the valley surrounded by mountains with peak heights between 2000 and 3000 m and side valleys of different size. The Inn Valley and its surroundings have been subject of various observational and numerical studies, exploring gap flows (Mayr et al. 2007), foehn winds (Gohm et al. 2004; Gohm and Mayr 2004), air pollution scenarios (Gohm et al. 2009; Schicker and Seibert 2009; Schnitzhofer et al. 2009), and the daytime up-valley flow, which is quite robust to synoptic forcing (Vergeiner and Dreiseitl 1987; Zängl 2004, 2009). A strong thermally-driven circulation in the Inn Valley also influences the velocity field at larger scales in the Bavarian foreland known as Alpine pumping. Observations and regional climate simulations suggest that this phenomenon occurs on average around 60 days per year (Graf et al. 2016).

### Observations

The so-called “Innsbruck Box” (i-Box) project is designed as a “reference box” to explore the ABL structure and exchange processes in complex terrain (Rotach et al. 2017). The i-Box observations consist of state-of-the-art measurement systems and instruments such as turbulence-flux towers (Stiperski and Rotach 2016) located in the Inn Valley, a scintillometer, a Doppler wind lidar, and a HATPRO temperature and humidity profiler (Massaro et al. 2015), located in the city of Innsbruck, and automatic weather stations in an extended mesonet.

**Fig. 1:**
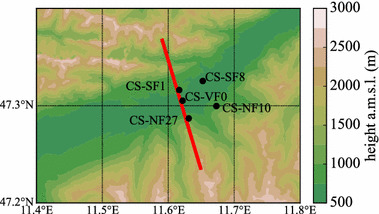
The i-Box stations (location indicated by points), with topography ($\Delta x=100\,\hbox {m}$, derived from the ASTER dataset; isolines every 250 m in the vertical) of the Inn Valley. For more information on the individual stations see Rotach et al. (2017). The red line denotes the cross-section in Fig. 3

We mainly focus on observations from the core sites (turbulence flux towers) located some 30 km east of the city of Innsbruck. Figure 1 shows the spatial distribution of the i-Box stations. The locations of the stations are representative for characteristic surfaces in complex terrain, such as the valley floor, and both south- and north-facing slopes. Since most of the flux towers are operational since the year 2013, this is a unique data pool of high-quality turbulence measurements, especially for locations in complex terrain, and offer the possibility to evaluate high-resolution NWP models in detail.

All the data analysis and quality control procedures of the measurement data are described in Stiperski and Rotach (2016). More specifically, the fluxes were calculated for 30-min averaging periods and double rotation was used to align the data into the slope-normal coordinate system. Prior to calculation the data were filtered using a recursive filter with a time constant of 200 s. Turbulence dissipation was estimated from the power spectra of the two horizontal velocity components using the spectral method (Piper and Lundquist 2004). For this purpose the inertial subrange was detected as the region where the power spectrum had a $-\,5/3$ slope. The 4/3 ratio between the power spectra of the horizontal velocity components was used as a quality criterion.

### Numerical Model

We perform numerical simulations with the COSMO model (version 5.0). The COSMO model is a limited area model that was originally developed for high-resolution, convection-resolving, operational NWP by the Deutscher Wetterdienst (Baldauf et al. 2011). Multiple national weather services have joined the consortium with their own versions of the model. Besides operational versions, simulations were also successfully performed for research purposes over mountainous terrain for, e.g., model evaluation studies with ABL observations (Collaud Coen et al. 2014), idealized model inter-comparison studies (Schmidli et al. 2010; Buzzi et al. 2011), and parametrization testing (Anurose and Subrahamanyam 2015; Panosetti et al. 2016). In our work, we focus on the evaluation of the operational set-up of MeteoSwiss with $\Delta x=1.1\,\hbox {km}$.

#### Set-up

The model solves the non-hydrostatic, fully compressible hydro-thermodynamical equations on an Arakawa C-grid. A third-order Runge–Kutta scheme is employed for time integration (Klemp and Wilhelmson 1978; Wicker and Skamarock 2002) and a fifth-order advection scheme is used for temperature, pressure, and velocity, while a second-order advection scheme is applied for moist quantities (Bott 1989). Radiation is parametrized via a $\delta$ two-stream radiation scheme (Ritter and Geleyn 1992), which includes a full cloud-radiation feedback. The effects of topographic shading are implemented in the model code following Müller and Scherer (2005), while a cumulus parametrization scheme after Tiedtke (1989) is switched on for shallow convection. The COSMO model uses the multi-layer soil model TERRA-ML consisting of eight soil levels with eight soil types. In the operational set-up, turbulence is parametrized with a 1.5-order closure following Mellor and Yamada (1982) with a prognostic equation for TKE. The model also offers an option to include horizontal shear production with a Smagorinsky-type turbulence treatment. More details can be found below.

**Fig. 2:**
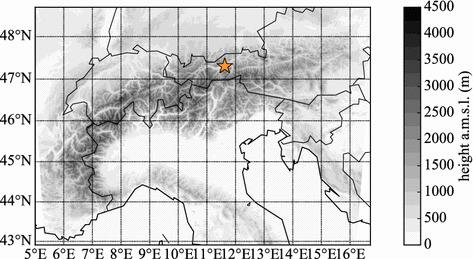
The inner domain of the COSMO model with the height of the model topography shown as grey contours (the contour interval is 250 m). The orange star marks the location of the area of interest

Our model set-up is similar to the operational COSMO-1 set-up of MeteoSwiss (de Morsier et al. 2012). The model uses two domains: the outer domain with a horizontal grid spacing of $\Delta x =6.6\,\hbox {km}$ (COSMO-7) spans Europe and is driven by ECMWF IFS-HRES data.^1^ The inner domain, which is slightly smaller than the operational domain by MeteoSwiss, consists of $800 \times 600$ grid points, spans the main Alpine range (Fig. 2), and uses the data from the outer domain as boundary fields. The horizontal grid spacing is $\Delta x = 1.1\,\hbox {km}$ with a timestep of $\Delta t=10\,\hbox {s}$, with 80 vertical levels employed in terrain-following smooth level vertical coordinates (Schär et al. 2002; Leuenberger et al. 2010). The lowest model half-level is located at a height of 10 m above ground and 40 vertical levels lie below 3000 m, which is roughly the height of the surrounding topography. The model topography (Fig. 2) is derived from the Advanced Spaceborne Thermal Emission and Reflection Radiometer (ASTER) Global Digital Elevation Map^2^ and resolves the Alps and their major valleys, including the Inn Valley, adequately for daytime up-valley flows (Zängl 2004, 2009). Closer comparisons with the high-resolution ASTER topography suggest that mountaintops are smoothed and that smaller side valleys are not well-resolved (Rotach et al. 2017, their Fig. 1), which may pose a challenge for the simulation of smaller-scale circulation patterns. External parameters for land-use and soil data on a horizontal grid spacing of $\Delta x= 1.1\,\hbox {km}$ are derived from the Harmonized World Soil Database (HWD^3^).

### Turbulence Parametrization Evaluation

We evaluate the model’s turbulence parametrization by means of case studies. The model is either initialized at 0000 UTC for daytime or 1200 UTC for night-time simulations and runs for 24 h; thus the first few hours of the simulations are considered as spin-up time and not considered in the analysis. We focus on days when the thermally-driven circulation dominates the ABL structure in the Inn Valley. Favorable weather patterns for these flows are cloud-free days, a strong up-valley flow in the Inn Valley (wind speed $> 4\,\hbox {m}\, \hbox {s}^{-1}$) and the corresponding wind direction ($\approx 090^{\circ }$ at the valley-floor station). During the consecutive cloud-free nights, drainage flows are present at the valley floor and on the slopes. Overall, we choose eight cases from the i-Box data pool that satisfy our criteria (simulation initiation time in brackets): 11 June 2014 (1200 UTC), 16 September 2014 (0000 UTC), 1 July 2015 (0000 and 1200 UTC), 28 August 2015 (1200 UTC), 29 August 2015 (0000 UTC), and 8 September 2015 (0000 and 1200 UTC). For all days, we conduct the simulations with both a 1D turbulence parametrization and a hybrid turbulence parametrization (see below).

The main quantity investigated is the TKE and its contributing budget terms, since it provides direct information about the status of the ABL (Stull 1988). When comparing the model to the measurements (Table 1), one must keep in mind that, (i) the height above mean sea level (a.m.s.l.) between model terrain and real terrain is different, (ii) the lowest model half-level is located at 10 m above ground level (a.g.l.), while the sensor heights of the flux towers are different, and (iii) the type of location (i.e., south-facing slope, valley floor) of the closest grid point of the model may diverge from the actual location, especially when the terrain representation is not appropriate.

Table 1 shows the height and slope angle of the i-Box stations and their representation in the model. The station on the valley floor (CS-VF0) is well-represented in terms of height a.m.s.l., however, in the model, the valley floor is slightly inclined. The stations with the optimun local terrain representation are on two slopes directly facing each other, namely CS-SF8 and CS-NF10. The other two stations on the slopes have either a too-steep slope angle in the model (CS-SF1) or a too-flat slope (CS-NF27). The type of location does not differ much, e.g., a north-facing slope is a north-facing slope in both observations and model.

**Table 1:** A detailed overview of the i-Box stations (see Fig. 1 for their locations) taken into account, and their representation in the numerical model: $\alpha$ indicates the slope angle

Station	Station height a.m.s.l. (m)	Model height a.m.s.l. (m)	Sensor height a.g.l. (m)	$\alpha \, (^{\circ })$	Model $\alpha \, (^{\circ })$
CS-VF0	545	577	8.7	0	2
CS-SF1	829	701	6.6	1	8
CS-SF8	575	556	11.4	8	7
CS-NF27	1009	853	6.8	27	15
CS-NF10	930	882	7.0	10	11

Since one single grid point of the model might not be representative of the ABL structure in that part of the valley, we employ a so-called grid-point ensemble: first, the closest model grid point to an i-Box station is determined via horizontal Euclidean distance. As a next step, we include the eight nearest grid points and calculate from this small nine-member ensemble the mean, median, 75th and 90th percentiles. Clearly, the question of how many members the grid-point ensemble should contain is related to the scale of the problem. Based on test simulations the scale of horizontal inhomogeneity is on the order of several 100 m–2 km while the scale of the topography is several km. The choice of nine ensemble members therefore is a compromise between these two constraints.

#### Observed TKE

When comparing the full TKE budget equation to that of the model, we have to consider the differences between modelled TKE budget terms and observed TKE budget terms. The observed TKE, $\bar{e}$, is directly calculated from the velocity variances observed at the i-Box stations

1 $$\begin{aligned} \bar{e}= \frac{1}{2} (\overline{u'^2}+\overline{v'^2}+\overline{w'^2}). \end{aligned}$$

Generally, the full TKE budget equation can be written as follows (Stull 1988)

2 $$\begin{aligned} \underbrace{\frac{ \partial \bar{e}}{ \partial t}}_\text {local tendency}+\underbrace{\overline{U_j}\frac{ \partial \overline{e}}{ \partial x_j}}_\text {advection}= & {} \underbrace{{\delta _{i3} \frac{g}{\overline{\theta _v}}(\overline{u_i'\theta _v'})} }_{\begin{array}{c} \text {buoyancy}\\ \text {production/consumption} \end{array}} -\underbrace{{\overline{u_i'u_j'}\frac{ \partial \overline{U_i}}{ \partial x_j}}}_{\begin{array}{c} \text {shear}\\ \text {production} \end{array}} \nonumber \\&-\underbrace{\frac{ \partial (\overline{u_j'e})}{ \partial x_j}}_\text {turbulent transport }- \underbrace{\frac{1}{\overline{\rho }} \frac{ \partial (\overline{u_i'p'})}{ \partial x_i}}_\text {pressure correlation}-\underbrace{{\varepsilon }}_\text {dissipation} \end{aligned}$$

where capital letters with overbars denote mean quantities, while small letters with primes refer to turbulent fluctuations; $\bar{e}$ is TKE, *U* is the mean wind speed, *g* is the acceleration due to gravity, $\theta _v$ is virtual potential temperature, $\rho$ is air density, and *p* is pressure. On the left-hand side (l.h.s.) are the local TKE tendency and advection with the mean flow, while on the right-hand side (r.h.s.) we find the thermally-driven buoyancy production/consumption term, the mechanical shear production term, the turbulent transport, the pressure correlation term, which mainly serves as TKE redistribution, and the TKE dissipation rate $\varepsilon$, which is always a sink for TKE.

#### 1D Turbulence Parametrization

While the full TKE equation is three-dimensional, the model’s turbulence closure considers only vertical turbulent processes. Note that this is the case for virtually all operational NWP settings—even when the model is operated at comparably high resolution and over complex terrain. In the first set of simulations, we therefore use the model’s 1.5-order turbulence closure at a 2.5-hierarchy level of Mellor and Yamada (1982), and refer to it as “turb_1D scheme”. The model solves the prognostic equation for the TKE making use of an auxiliary variable *q* defined by $q=\sqrt{2\bar{e}}$ or vice versa $\bar{e}={q^2}/2$,

3 $$\begin{aligned} \underbrace{\frac{D}{Dt}\left( \frac{q^2}{2}\right) }_\text {tendency}= & {} -\underbrace{K_H \frac{g}{\theta } \frac{\partial \theta }{\partial z}}_{\begin{array}{c} \text {buoyancy} \\ \text {production/consumption} \end{array}} + \underbrace{ K_M\left[ \left( \frac{ \partial U}{ \partial z}\right) ^2+\left( \frac{ \partial V}{ \partial z}\right) ^2\right] }_\text {vertical shear production} \nonumber \\&+\underbrace{\frac{1}{\overline{\rho }}\frac{ \partial }{ \partial z}\left[ \alpha _{\text {tke}}\overline{\rho }\lambda _l q \frac{ \partial }{ \partial z}\left( \frac{q^2}{2}\right) \right] }_\text {vertical turbulent transport}-\underbrace{\frac{q^3}{B_1\lambda _l}}_\text {dissipation}. \end{aligned}$$

The term on the l.h.s. is the tendency of TKE. Note that this term can be split into a local tendency part ($\frac{\partial }{\partial t} \frac{q^2}{2}$) and an advection part. However, the advection is only invoked in the hybrid turbulence parametrization as described in the follow-up section. The first two terms on the r.h.s. are the buoyancy production/consumption term and the vertical shear production term, where $K_H$ and $K_M$ are the turbulent diffusivity and conductivity, respectively. For more details on the calculation of stability functions and constants see Appendix B-1 of Buzzi (2008) and Buzzi et al. (2011). In the vertical turbulent transport term, $\alpha _{\text {tke}}$ is a parameter from the parametrization scheme, and the turbulence length scale $\lambda _l$ is calculated after Blackadar (1962),

4 $$\begin{aligned} \lambda _l=\lambda ^{\infty }_{l}\frac{\kappa (z+z_0)}{\kappa (z+z_0)+\lambda ^{\infty }_{l}}. \end{aligned}$$

Here, $\lambda ^{\infty }_{l}$ is an asymptotic length scale, $z_0$ is the aerodynamic roughness length, $\kappa$ is the von Kármán constant, and *z* is the respective height above ground. The Blackadar length scale is a widely-used approach and was developed for larger scales, though it should be noted that it might perform poorly in convective conditions when eddies reach the size of the ABL height (Chrobok et al. 1992).

We note here that the pressure correlation term (Eq. 2) plays a similar role as the turbulent transport term in redistributing TKE. Since it is usually considered small it is not explicitly modelled in the operational turb_1D scheme. In complex terrain, however, taking the pressure correlation term to be small may be a dangerous assumption. The dissipation follows Kolmogorov’s law, while $B_1$ is a constant model parameter after Mellor and Yamada (1982). Note that this parametrization of the dissipation rate implies isotropic turbulence, which is often not the case in shear-generated turbulence in complex terrain. The COSMO model has also an option to include subgrid-scale orographic drag. However, at our horizontal grid spacing, the larger-scale terrain is already adequately resolved, therefore this effect is considered as small and is consequently not invoked; however, it could play a role in the stable ABL (Steeneveld et al. 2009).

The model’s surface transfer scheme uses a diagnostic TKE equation (Buzzi 2008, Appendix B-2). The surface transfer layer is defined as the layer between the surface and the lowest model level, where the transfer coefficients are computed, and consists of three sublayers: a laminar sublayer, a turbulent roughness sublayer, and a constant-flux (Prandtl) sublayer. The roughness sublayer reaches from the surface, where the turbulent length scale $l=\lambda /\kappa$ is zero, to a level where $l=z_0$. The Prandtl sublayer above extends from *l* up to the first model level, therefore a discrimination between model variables at the surface predicted by the soil model and atmospheric values at *l* is possible. The fluxes are formulated in resistance form and interpolation schemes are used for the calculation of the transport resistances. The necessary boundary-layer profiles are derived from the dimensionless coefficients $K_M$ and $K_H$ of the Mellor–Yamada framework. This scheme therefore avoids the empirical functions of Monin-Obukhov similarity theory, which are in general not applicable in complex terrain (Stiperski and Rotach 2016). This surface transfer scheme is employed in the operational set-ups of the COSMO model (Baldauf et al. 2011), and also in the regional climate model version, COSMO-CLM (Langhans et al. 2013; Leutwyler et al. 2016).

#### Hybrid Turbulence Parametrization

Extensions to the turb_1D scheme are available in the COSMO model (Blahak 2015): the advection of $q=\sqrt{2 \bar{e}}$ is computed together with the other advective tendencies of model variables and added in the follow-up timestep to the TKE budget. Recall that in the model code, the TKE equation is solved for $q=\sqrt{2 \bar{e}}$, because *q* is an important quantity in the Mellor–Yamada framework. Therefore, the advected quantity is *q*, not directly the TKE, $\bar{e}$. Horizontal diffusion is calculated in terrain-following coordinates.

The vertical shear production term in the TKE equation can be extended to three dimensions, thereby also considering horizontal shear production in the TKE budget. This is achieved by calculating the horizontal contributions to shear production with a Smagorinsky closure (Smagorinsky 1963; Langhans et al. 2012). The shear production of TKE due to horizontal gradients is determined as follows and then added to the model TKE equation,

5 $$\begin{aligned} \frac{\partial }{\partial t}\left( \frac{q^2}{2}\right) _{{Shear}_{hor}}=(c \Delta x)^2 \left[ \left( \frac{ \partial U}{ \partial x}\right) ^2+\left( \frac{ \partial V}{ \partial y}\right) ^2+\frac{1}{2}\left( \frac{ \partial U}{ \partial y}+\frac{ \partial V}{ \partial x}\right) ^2\right] ^{\frac{3}{2}}, \end{aligned}$$

where *c* is the dimensionless Smagorinsky constant with a value of 0.2, and $\Delta x$ is the horizontal grid spacing.

With these two additional contributions to the TKE equation, we have a “hybrid” set-up (“turb_hybrid” scheme hereafter): the vertical contributions to the TKE is calculated with the Mellor–Yamada framework, while the horizontal contributions to TKE are calculated with a method usually used for large-eddy simulations (LES). The COSMO model also offers a fully 3D Smagorinsky-Lilly scheme for LES, which is not suitable for our horizontal grid spacing (Honnert and Masson 2014; Cuxart 2015).

#### Comparison to Observations

It is challenging to compare all the terms of the TKE budget to the available observations, therefore we provide an overview of our methodology in the following paragraphs.

We have observations of TKE itself, the buoyancy production/consumption term (estimated with the observed sensible heat flux), and the TKE dissipation rate. Therefore, these quantities can be directly compared to the model results from the lowest model level. The observed vertical turbulent transport can be approximated for stations with TKE observations at two or more levels, which is the case at the valley floor station (CS-VF0),

6 $$\begin{aligned} \frac{ \partial (\overline{u_3'e})}{ \partial x_3}\approx \frac{\Delta (\overline{w'e})}{\Delta z} \end{aligned}$$

where the values for the difference are taken from the lowest (4 m) and highest (17 m) level of the measurement tower. Similarly, vertical shear production can be estimated from flux towers with two or more levels of mean wind observations (CS-VF0, CS-NF27, and CS-SF8), and can be compared with the model’s vertical shear production term. We estimate four horizontal terms of the shear production (Eq. 2) at the valley floor station (CS-VF0) with the observations from the south-facing slope station that is located at a similar altitude (CS-SF8, Fig. 1),

7 $$\begin{aligned}&\overline{u'u'}\frac{ \partial \overline{U}}{ \partial x}+ \overline{u'v'}\frac{ \partial \overline{U}}{ \partial y}+ \overline{u'v'}\frac{ \partial \overline{V}}{ \partial x}+ \overline{v'v'}\frac{ \partial \overline{V}}{ \partial y} \nonumber \\&\quad \approx \overline{u'u'} \frac{\Delta \overline{U}}{\Delta x}+\overline{u'v'} \frac{\Delta \overline{U}}{\Delta y}+\overline{u'v'} \frac{\Delta \overline{V}}{\Delta x}+\overline{v'v'} \frac{\Delta \overline{V}}{\Delta y} \end{aligned}$$

where the velocity components $\overline{U}$ and $\overline{V}$ are rotated back into the Cartesian coordinate system, and $\Delta x=2.19\, \hbox {km}$ and $\Delta y=2.25\, \hbox {km}$, respectively. Here the fluxes are taken from the CS-VF0 station, noting that there are no observations of mean TKE advection.

## Results

### 1D Turbulence Parametrization

In the following sections, we present a typical daytime (1 July 2015, simulation started at 0000 UTC) and a typical night-time situation (1 July 2015, simulation started at 1200 UTC) in the Inn Valley with cross-sections, vertical profiles, and time series from the model run with the turb_1D scheme.

**Fig. 3:**
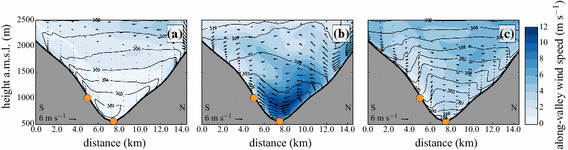
Interpolated along-valley wind speed (colour contours) on a south–north cross-section along the red line in Fig. 1 for **a** 1000 UTC, **b** 1500 UTC, and **c** 2400 UTC on 1 July 2015. Wind arrows are calculated from the cross-valley component *v* and the vertical velocity component *w*, respectively. Black contours indicate isentropes (intervals: 3 K). Orange dots mark the location of the two i-Box stations CS-VF0 and CS-NF27. “S” and “N” indicate south and north, respectively

#### Cross-Sections

Figure 3 shows the cross-valley circulation on a south-north cross-section on 1 July 2015 at three different times (1000 UTC, 1500 UTC, and 2400 UTC). Shortly after sunrise, upslope flows are established at both the north- and south-facing slopes (Fig. 3a), and prevail for several hours. A mixed layer forms at the valley floor, while along-valley wind speeds remain below $2\, \hbox {m}\, \hbox {s}^{-1}$. The isentropes, steepening on the slopes, indicate the growing mixed layer at the valley floor, the slope-flow layers on the two slopes, and the stable layer aloft.

This situation is different at 1500 UTC (Fig. 3b), when a strong up-valley flow dominates the ABL structure in the Inn Valley. The upslope flows are largely eroded and a strong cross-valley circulation is present. The afternoon up-valley flow with wind speeds up to $12\, \hbox {m}\, \hbox {s}^{-1}$ is not disturbed by the cross-valley circulation and the downslope flows on the south-facing slope. Note the asymmetric structure of the up-valley flow, where the south-facing slope experiences higher wind speeds ($> 10\, \hbox {m}\, \hbox {s}^{-1}$) than the north-facing slope ($4\, \hbox {m}\, \hbox {s}^{-1}$). The asymmetry in the up-valley flow is likely due to the curvature of the Inn Valley at this specific location; similar curvature effects were also reported in Weigel and Rotach (2004) for the north-south oriented Riviera Valley.

After sunset, the up-valley flow breaks down and downslope flows are established on the slopes, which is also visible in the isentropes on the slopes. Low wind speeds around $2\, \hbox {m}\, \hbox {s}^{-1}$ dominate along the valley and at the slopes, and a stable ABL forms at the valley floor (Fig. 3c).

**Fig. 4:**
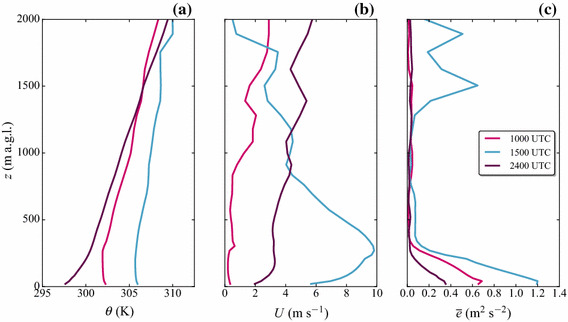
Modelled vertical profiles of **a** potential temperature, **b** horizontal wind speed $U=\sqrt{u^2+v^2}$, and **c** TKE at the grid point nearest to the valley floor station CS-VF0 at 1000 UTC, 1500 UTC, and 2400 UTC on 1 July 2015

#### Vertical Profiles

Vertical profiles from model output of the valley floor station (CS-VF0) are presented in Fig. 4 for 1000 UTC, 1500 UTC, and 2400 UTC. At 1000 UTC, we find a developing mixed layer near the ground in the potential temperature profile, capped by a weak inversion at approximately 250 m above ground. Above this mixed layer, the atmosphere is stably stratified. The wind speeds are still low ($<1\, \hbox {m}\, \hbox {s}^{-1}$), while TKE values are highest close to the ground with a maximum of around $0.6\, \hbox {m}^{2} \, \hbox {s}^{-2}$. In the afternoon, the up-valley flow clearly dominates the ABL structure at the valley floor, with the model suggesting a strong jet-like velocity maximum (up to $10\, \hbox {m}\, \hbox {s}^{-1}$) at around 250 m above ground. Together with the strong up-valley flow, a near-ground TKE maximum is present, while there are secondary TKE maxima visible at higher altitudes, also observed in the Rivera Valley (Weigel et al. 2007). In the night a stable ABL is established, evident in the corresponding potential temperature profile. A weak, jet-like velocity structure near the ground suggests down-valley drainage flows at the valley floor, associated with smaller TKE values ($< 0.6\, \hbox {m}^{2} \, \hbox {s}^{-2}$) compared to the daytime ($> 1\, \hbox {m}^{2} \, \hbox {s}^{-2}$).

#### Time Series: Day

**Fig. 5:**
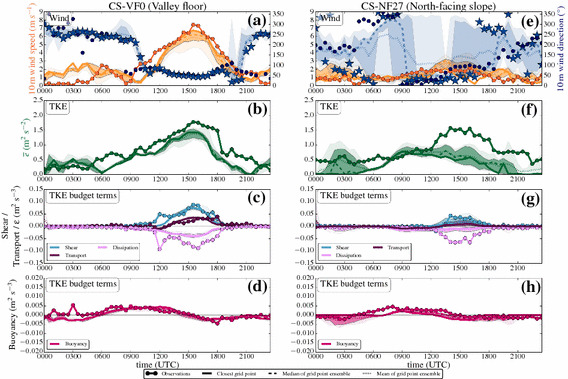
Time series of **a**, **e** wind speed and wind direction, **b**, **f** TKE, and TKE budget terms (**d**, **h** buoyancy, **c**, **g** vertical shear, **c**, **g** vertical turbulent transport, and **c**, **g** dissipation) of 1 July 2015 (started at 0000 UTC, daytime simulation), from both observations (from level 2, if not otherwise indicated), and model output (from the lowest model level at 10 m) for two i-Box locations: the valley floor (CS-VF0, **a**–**d**) and north-facing slope (CS-NF27, **e**–**h**). Filled dots indicate observations, while bold solid lines (or in the case of wind direction, stars) denote the model output from the closest grid point, the dotted lines show the arithmetic mean, the dashed lines show the median, and the shaded areas surrounding the median indicate the 75th (dark colours) and 90th (light colours) percentiles, of the grid-point ensemble, respectively. Colours refer to the variables on the coloured axes (**a**, **b**, **d**, **e**) and are given in the legend for the TKE budget terms (**c**, **f**, **g**, **h**). Turbulence closure for the simulations is the turb_1D scheme

Figure 5 shows time series of wind speed, wind direction, TKE, and the TKE budget terms over the whole simulation period of 24 h on 1 July 2015, together with the observations for the same time period from both the station on the valley floor (CS-VF0) and the north-facing steep slope (CS-NF27). The first few hours of simulation are considered as model spin-up time, during which the model adjusts to the observed, relatively weak down-valley drainage flow in the centre of the valley (Fig. 5a) and to a downslope flow on the north-facing slope (Fig. 5e).

Immediately after sunrise (around 0400 UTC), upslope flows develop on the north-facing slope (Fig. 5e). This change in wind direction is also simulated by the model, although with a time delay of two hours, which might be related to the model topography representation. During the development of the mixed layer in the valley, wind speeds generally remain small in both the model and observations. TKE starts to increase after sunrise at both the valley floor and the north-facing slope (Fig. 5b, f), where the main TKE production mechanism during this time is buoyancy production (Fig. 5d, h). During the time period before noon, TKE is well simulated, together with a satisfying representation of the buoyancy production term, which reaches its daytime maximum between 0900 UTC and 1200 UTC.

Together with the increase of wind speed, an increase in TKE is observable. The up-valley flow establishes at around 1100 UTC with a change in wind direction to $\approx 090^{\circ }$ at the CS-VF0 station (Fig. 5a). The up-valley flow gains strength until 1500 UTC with wind speeds reaching $> 7\, \hbox {m}\, \hbox {s}^{-1}$ at the valley floor. In both the model and measurements, the wind-speed maximum is synchronous with the afternoon TKE maximum together with a maximum in vertical shear production (Fig. 5c, g). Although the vertical shear production is modelled adequately, TKE is still underestimated by the model.

The model is able to simulate the spatial variability between the stations: at the CS-NF27 station, the up-valley flow is not as well-established, but erodes the upslope flows, visible in the change of wind direction during the early afternoon. Vertical shear production is correctly simulated at both locations, but at the CS-NF27 station the modelled TKE is significantly underestimated by about $1\, \hbox {m}^{2} \, \hbox {s}^{-2}$ (Fig. 5f). Since the dissipation rate is smaller in the model than in the observations, we assume that the model is not overly diffusive. In the late afternoon, the up-valley flow weakens together with a decrease in TKE until the flow finally breaks down after 1800 UTC.

#### Time Series: Night

**Fig. 6:**
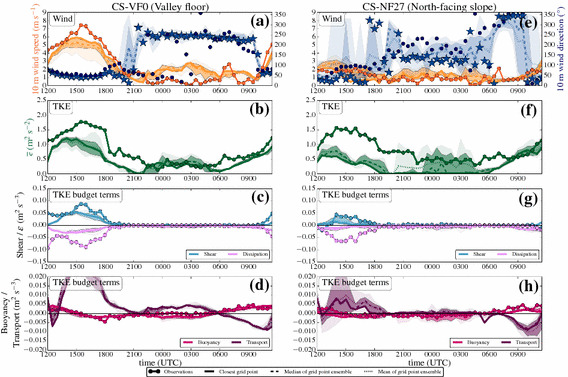
As in Fig. 5, but for 1 July 2015, started at 1200 UTC (night-time simulation). Note that the vertical turbulent transport is now plotted on the same axis as buoyancy production. Turbulence closure for the simulations is the turb_1D scheme

Figure 6 shows the corresponding time series of wind speed and direction, TKE, and the associated budget terms during the follow-up night. After sunset and the evening transition, the up-valley flow finally breaks down, and according to the simulation, a down-valley drainage flow establishes at the CS-VF0 station (Fig. 6a). The model suggests a constant wind direction of $\approx 250^{\circ }$, while the observations show highly varying wind directions, as is quite common for low wind speeds. However, the model overestimates the wind speed of the down-valley drainage flow, which is also reflected in the TKE (Fig. 6b). The closest grid point in the model to the CS-VF0 station has a small inclination compared to the nearly flat real terrain, which may enhance the flow (Wagner et al. 2015b). Larger modelled TKE values than observed occur at the same time as the overestimated wind speeds (2200–0500 UTC). In contrast to the observations, the buoyancy production term is mainly negative in the model (Fig. 6d). The local vertical turbulent transport plays an important role on the valley floor, especially in counter-acting the buoyancy consumption, while the other budget terms are almost zero.

The CS-NF27 station is mostly influenced by downslope flows during the night according to the dominating wind direction ($120^{\circ }$–$200^{\circ }$) in both model and observations (Fig. 6e). Differences in wind direction are partly related to the different slope angles in the model terrain and in reality: the slope angle in reality is substantially larger ($27^{\circ }$) than in the model ($15^{\circ }$). Terrain steepness is a major parameter for the correct simulation of slope flows (Wagner et al. 2015a). After sunset observed TKE generally decreases to smaller values than during the daytime, although a minimum TKE $\approx 0.5 \, \hbox {m}^{2} \, \hbox {s}^{-2}$ remains during the night. The model is not successful in simulating the night-time TKE at all (Fig. 6f): after the evening transition, TKE values close to zero are simulated, and this basically remains throughout the whole night, while the model is not capable of producing any TKE during this time period. The modelled buoyancy consumption, vertical shear production, and dissipation rate of TKE closely match the observations during this time with all involved production terms around zero (Fig. 6g, h). However, several of the surrounding closest grid points in the ensemble suggest TKE values > zero, even if at the same time the vertical turbulent transport term at the closest grid point is also near zero.

### Hybrid Turbulence Parametrization

In the following two sections, we present time series and vertical profiles of the same case study day (1 July 2015), but now from the simulation with the turb_hybrid scheme. We also provide an estimate of the horizontal shear production from observations.

#### Time Series: Day

**Fig. 7:**
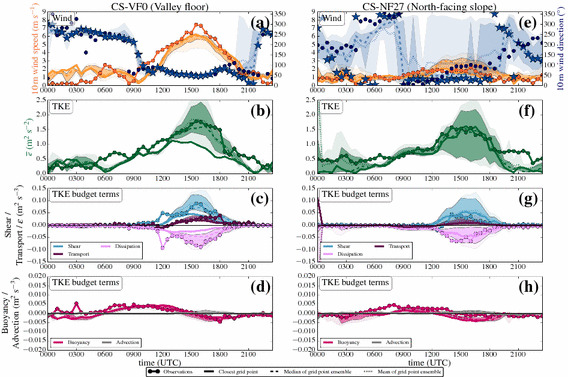
As in Fig. 5, but with the turb_hybrid scheme. Time series of **a**, **e** wind speed and wind direction, **b**, **f** TKE, and TKE budget terms (**d**, **h** buoyancy, **c**, **g** 3D shear, **c**, **g** vertical turbulent transport, **c**, **g** dissipation, and **d**, **h** TKE advection)

Figure 7 shows the results for 1 July 2015 when invoking the turb_hybrid scheme with the horizontal shear production and TKE advection. Compared to the simulation with the turb_1D scheme, the model does not exhibit much change in wind speed and direction at the CS-VF0 station (Fig. 7a). For the CS-NF27 station, the upslope flows now start earlier (0400 UTC) in the model (Fig. 7e). The new 3D effects do not have a large influence on the simulation of TKE before noon when buoyancy is the main production mechanism.

The up-valley flow is characterized by strong horizontal shear production, which is visible in Fig. 7c: together with the now 3D shear production term, the TKE structure in the model becomes more realistic. The median of the TKE of the grid-point ensemble (Fig. 7b) is in much better agreement with the observations compared to the turb_1D scheme (Fig. 5b).

Note, however, that the nearest grid point does not follow the general improvement (on the contrary). Inspecting the spatial TKE distribution at the time of largest discrepancy (1600 UTC) reveals that the nearest grid point coincidentally is the one with smallest TKE within the grid-point ensemble. Also, there is a strong positive skewness in the distribution of TKE values (not shown) so that indeed, even with a nine-member ensemble, one member (which happens to be the nearest grid point) can fall out of the 90th percentile. This fact emphasizes the added value of the grid-point ensemble: the model seems to be able to simulate the TKE budget appropriate at scales larger than the horizontal grid spacing. However, the model cannot be expected to capture the very local spatial gradients, therefore the TKE values at one single grid point might be underestimated.

The maximum of the up-valley flow, the TKE maximum, and the shear production maximum occur simultaneously at the CS-VF0 station. The panels c) and g) now show the full 3D shear production from the simulation while only vertical shear production is available from the observations (see Sect. 3.2.2 for a rough estimate of horizontal shear production from the measurements).

On the north-facing slope, we find an almost perfect simulation of TKE during both morning and the afternoon (Fig. 7f), together with a realistic simulation of the budget terms (Fig. 7g, h). The second 3D effect, TKE advection, is small throughout the whole simulation at both stations and seems to have a minor influence on the TKE structure during daytime.

#### Horizontal and Vertical Shear Production from Observations

**Fig. 8:**
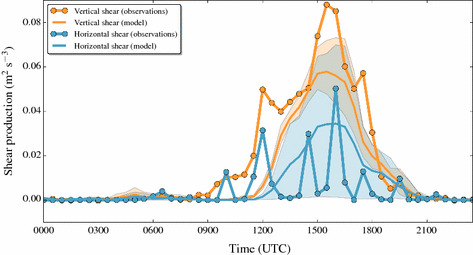
Time series of horizontal shear production (blue) and vertical shear production (orange) at the station CS-VF0 of 1 July 2015 (started at 0000 UTC, daytime simulation), from both observations (lines with dots) and the mean of the grid-point ensemble of the model output (full bold lines). Note that the observed horizontal shear production includes the estimate of four terms (Eq. 7) with rotated velocity components and velocity variances

A comparison of the shear production terms with observations is somewhat difficult since the vertical contribution is locally estimated while the horizontal contributions are a combination of a very crude horizontal wind-gradient approximation between two neighbouring sites and the local fluxes at one site (Sect. 2.4.4, Eq. 7). In Fig. 7, therefore, observed shear production only includes the vertical term (which is the only one present in the turb_1D scheme). As we have only very approximate estimates of the horizontal shear production terms we show them separately in Fig. 8, using the daytime simulation of 1 July 2015. The model and the observations agree that vertical shear production is a substantial source of TKE in the afternoon—although a significant underestimation has to be noted especially around the time of the peak. Modelled horizontal shear production shows a smoother (and likely more realistic) time series than the observations, the high-frequency variability of which points to the large uncertainty in their estimation. However, the order of magnitude between model and observations is similar, and the peaks in estimated horizontal shear production occur mostly in the afternoon together with the peak of observed vertical shear production. The observations show that horizontal shear production is not only present in the numerical simulation, but also an important observable TKE production term at the valley floor station, which may be related to the afternoon maximum of the strong up-valley flow at this location (Fig. 3b). This is similar to Weigel and Rotach (2004), who also found strong horizontal gradients in wind speeds for the up-valley flow leading to shear production of TKE. The length scale of these exchange processes is related to the valley width.

**Fig. 9:**
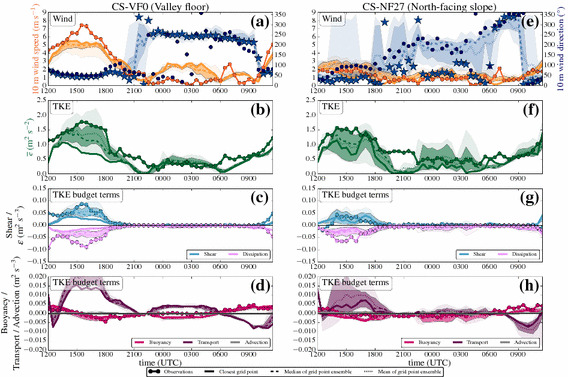
As in Fig. 5, but for 1 July 2015, started at 1200 UTC (night-time simulation) with the turb_hybrid scheme. Time series of **a**, **e** wind speed and wind direction, **b**, **f** TKE, and TKE budget terms (**d**, **h** buoyancy, **c**, **g** 3D shear, **d**, **h** vertical turbulent transport, **c**, **g** dissipation, and **d**, **h** TKE advection)

#### Time Series: Night

In Fig. 9, the results for the night-time simulation with the turb_hybrid scheme are presented. After the evening transition, the down-valley drainage flow is also present in the new simulation with 3D shear production (Fig. 9a). The TKE structure during the night is well-simulated at the valley floor, similar to the simulation with the turb_1D scheme. Sometimes, TKE is even overestimated, although buoyancy acts as a damping term. Vertical turbulent transport is an important local source contributing to the TKE budget during night-time and makes a somewhat larger contribution to the TKE budget in the turb_hybrid scheme. Note that the contribution of the vertical turbulent transport to the TKE budget has a major influence, while during the day plays a minor role for the overall TKE budget. The TKE structure at the valley floor is still strongly influenced by the down-valley drainage flow, which is overrepresented in the model.

At the CS-NF27 station, we find major improvements: after the evening transition, TKE reaches low values again (Fig. 9f); however, the model is now able to produce TKE during night-time, which is a major difference to the simulation with the turb_1D scheme. The surrounding grid-point ensemble even suggests values that are of the same magnitude as the observations. This is related to a similarly small vertical turbulent transport term as in the turb_1D scheme in conjunction with the simulation of stronger downslope flows (see next section). The stable ABL over the steep slope provides the only situation where TKE advection notably contributes to the TKE structure.

**Fig. 10:**
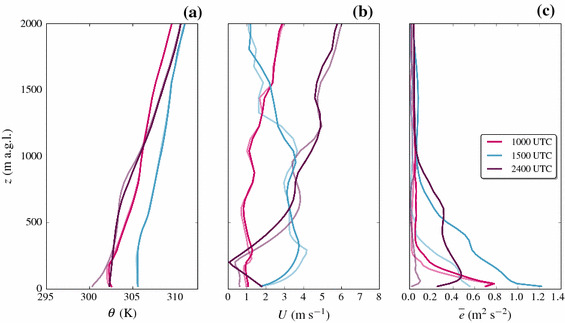
Modelled vertical profiles of **a** potential temperature, **b** horizontal wind speed $U=\sqrt{u^2+v^2}$, and **c** TKE at the nearest grid point to the north-facing slope station CS-NF27 at 1000 UTC, 1500 UTC, and 2400 UTC on 1 July 2015. The transparent lines show the profiles from the simulation with the turb_1D scheme, while the full lines show the vertical profiles from the simulation with the turb_hybrid scheme

#### Vertical Profiles

Figure 10 shows the impact of the turb_hybrid scheme on vertical profiles of potential temperature, horizontal wind speed, and TKE for the slope station (CS-NF27), because after the introduction of the turb_hybrid scheme the changes in ABL structure are more substantial than at the valley-floor station (CS-VF0). At 1000 UTC, when a mixed layer is present and buoyancy dominates the TKE production, almost no changes are found with the turb_hybrid scheme. In the afternoon, at 1500 UTC, the up-valley flow dominates the ABL structure in the valley. Compared to the turb_1D scheme, the jet-like structure of the horizontal wind speed is more smoothly simulated, while the afternoon TKE has significantly higher values also at some distance away from the surface. During the night, when a stable ABL is present, the model is able to simulate notable TKE with the turb_hybrid scheme. The resulting stratification changes from strongly stable in the lowest 300 m to only a weak stratification (Fig. 10a). Unfortunately, we do not have observed vertical temperature profiles at site CS-NF27 to judge whether the turb_hybrid scheme yields a more realistic—and not only a different—ABL structure. Inspecting the relation between dynamic stability *z* / *L* (where *z* is the respective height and *L* is the Obukhov length) and TKE near the surface, shows that indeed larger observed values of TKE are associated with reduced dynamic stability. The turb_1D scheme does not reproduce this behaviour (evaluated a the lowest model level) while the relation is reproduced by the turb_hybrid scheme (not shown). We therefore cautiously conclude that the hybrid TKE treatment improves not only the TKE itself, but also leads to a better representation of the vertical ABL structure.

## Validation for all Case Study Days

In this section, we validate the model performance with respect to TKE for all case studies (see overview in Sect. 2.4) and all i-Box sites (Fig. 1, Table 1) together. We distinguish three categories on the basis of the different boundary-layer turbulence forcings based on the dominant TKE source:

1. Buoyancy-driven TKE: the typical before-noon situation, when the buoyancy term is the main source of TKE production, a mixed layer forms at the valley floor (Fig. 4a, 1000 UTC), and upslope flows are present. As a criterion, we choose the time frame between 0500 and 1200 UTC, when the shortwave radiative flux is larger than zero, and wind speed is still below a $3\, \hbox {m}\, \hbox {s}^{-1}$ threshold (between 0600 UTC and 1130 UTC in Fig. 5b).
2. Shear-dominated TKE: this situation is present when the wind speed exceeds the threshold of $3\, \hbox {m}\, \hbox {s}^{-1}$, and the wind direction is around $090^{\circ }$ at the valley floor suggesting an up-valley flow (Fig. 4b). In this case the main source for TKE is shear production, which is dominant between 1200 and 1800 UTC (Fig. 5).
3. Transport-dominated TKE: the main criteria in this case are that the shortwave flux has reached zero, the wind direction corresponds to a down-valley drainage flow ($\approx 250^{\circ }$) on the valley floor, and to the corresponding slope-flow directions at the slope stations ($120^{\circ }$-$200^{\circ }$ for site CS-NF27, see Fig. 6), respectively. A stable ABL forms during night-time, which can also be seen in the vertical profiles (Figs. 4 and 10, 2400 UTC potential temperature profile). TKE values are generally lower than during daytime, while vertical turbulent transport is the major local TKE source.

The ${\textit{bias}}$ and root-mean-square error (${\textit{rmse}}$) are computed as in Chow et al. (2006),

8 $$\begin{aligned} bias=\frac{1}{N_s}\sum ^{N_s}_{j=1}\left( \frac{1}{N_t}\sum ^{N_t}_{i=1}{{M_{i}-O_i}}\right) \end{aligned}$$

and

9 $$\begin{aligned} rmse=\left[ \frac{1}{N_s}\sum ^{N_s}_{j=1}\left( \frac{1}{N_t}\sum ^{N_t}_{i=1}{(M_{i}-O_i)}^2\right) \right] ^\frac{1}{2}, \end{aligned}$$

where $M_i$ is the median of the grid-point ensemble, $O_i$ are the i-Box observations, $N_t$ is the number of timesteps of the chosen turbulence forcing (buoyancy-driven, shear-driven, or transport-driven) and $N_s$ is the number of i-Box stations taken into account. We chose the median of the grid-point ensemble instead of the closest grid point from the model output, because the median is a better measure to represent the ABL structure in the valley accordingly. ${\textit{Bias}}$ and ${\textit{rmse}}$ values are calculated separately for each TKE forcing type, taking all respective hours of the day into account, all available days and two groups of i-Box sites: valley floor (stations CS-VF0, CS-SF1, and CS-SF8; $N_s=3$), and north-facing slopes (stations CS-NF27 and CS-NF10, $N_s=2$). Although the stations CS-SF1 and CS-SF8 are located at a south-facing slope (also in the model topography), the wind structure in both observations and model exhibits a strong up-valley flow influence (not shown), which is similar to the wind time series of the valley floor station in the centre of the valley (CS-VF0).

**Table 2:** ${\textit{Bias}}$ and ${\textit{rmse}}$ values for TKE for simulations with both the turb_1D scheme and the turb_hybrid scheme

TKE forcing	Location	${\textit{bias}}$ [1D] ($\hbox {m}^{2}\, \hbox {s}^{-2}$)	${\textit{bias}}$ [hybrid] ($\hbox {m}^{2} \, \hbox {s}^{-2}$)	${\textit{rmse}}$ [1D] ($\hbox {m}^{2} \, \hbox {s}^{-2}$)	${\textit{rmse}}$ [hybrid] ($\hbox {m}^{2}\, \hbox {s}^{-2}$)
Buoyancy	Valley floor	$-$ 0.32	$-$ 0.30	0.36	0.34
	Slopes	0.03	0.04	0.16	0.15
Shear	Valley floor	$-$ 0.44	0.08	0.48	0.33
	Slopes	$-$ 0.45	$-$ 0.22	0.51	0.34
Transport	Valley floor	$-$ 0.22	$-$ 0.12	0.25	0.16
	Slopes	$-$ 0.35	$-$ 0.32	0.38	0.36

The values have been calculated from all performed simulations (8 in total) for buoyancy-driven TKE, afternoon shear-dominated TKE, and the turbulent-transport dominated night-time stable ABL. The location “valley floor” includes the i-Box stations CS-VF0, CS-SF8, and CS-SF1, while “slopes” include the i-Box stations CS-NF27 and CS-NF10

Table 2 summarizes the error statistics. First, with the turb_1D scheme, TKE is generally underestimated for all forcing types and locations. An exception is the buoyancy-forced situation on the slopes, suggesting the model is able to simulate the TKE structure successfully, when vertical turbulent exchange processes are dominant. In the simulations with the turb_hybrid scheme, we find a better simulation of TKE for all three ABL situations, although the degree of improvement is different: with buoyancy-driven TKE production, we find only small improvements of TKE representation. The turb_hybrid scheme has the highest impact on TKE during the up-valley flow phase (shear-dominated TKE): the ${\textit{bias}}$ for TKE decreases by more than $0.3\, \hbox {m}^{2}\, \hbox {s}^{-2}$ at the valley floor stations, and at the slope stations to values as small as 0.22. The simulation of the transport-dominated TKE during the night improves with the turb_hybrid scheme at the valley floor station, while on the slopes there are only minor improvements for ${\textit{bias}}$ and ${\textit{rmse}}$. This suggests that the turb_hybrid scheme has a positive impact on the TKE simulation, but challenges associated with the simulation of the stable ABL (especially on the slope) remain. Of course, we cannot rule out that the improved TKE simulation with the turb_hybrid scheme is (at least partly) due to compensating errors. Still, the case studies at two representative sites in Sect. 3 demonstrate that, in general, an improved simulation of TKE is associated with the additional budget terms considered in the turb_hybrid scheme, and that these additional terms are appropriately modelled. It is worth mentioning that at the second north-facing slope station (CS-NF10)—which is by far better represented in the model topography (Table 1)—the TKE is successfully simulated with both turbulence parametrizations. This suggests that correct terrain representation is also an important factor leading to a appropriate simulation of ABL structure.

## Discussion

The presented results show that the high-resolution NWP model COSMO-1 is able to adequately simulate the general ABL structure in the Inn Valley. We found similar features in model performance on all case study days: TKE is underestimated by the turb_1D scheme. Invoking 3D terms (turb_hybrid scheme) is beneficial in all case studies, especially during shear-dominated situations, in line with Stiperski and Calaf (2017) who show that when describing shear-driven turbulence two length scales (vertical and horizontal) are needed.

The terrain of the Inn Valley is sufficiently represented by the COSMO model with the exception of steeper slopes, mountaintops and smaller side valleys. As such, the model is successful in simulating the daytime up-valley flow at the valley floor in terms of wind speed and wind direction. During night-time, terrain-related features play a more important role, especially for the simulation of slope flows and the down-valley drainage flows.

The turb_1D scheme is able to simulate the major structure of the daytime ABL in the Inn Valley, such as the upslope flows, a growing mixed layer, and the up-valley flow. This is in agreement with Schmidli et al. (2010), who found in a model inter-comparison study that parametrizations of the Mellor–Yamada type are successful in simulating the thermally-driven circulation. On our investigated days, before noon when buoyancy is the main TKE production term, the TKE generation is dominated mostly by vertical exchange, which is well-simulated by the turb_1D scheme.

However, during shear-dominated up-valley flow phases, the turb_1D scheme is no longer sufficient; the TKE is underestimated by the model, although the general diurnal cycle of TKE is well-simulated. This is in agreement with Couvreux et al. (2016), who compared the results from two NWP models (ARPEGE, $\Delta x=9\, \hbox {km}$, and AROME, $\Delta x =2.5\, \hbox {km}$) with near-ground TKE observations during the BLLAST campaign in a complex and inhomogeneous environment north of the Pyrenees. While the ARPEGE model mostly simulated the diurnal TKE cycle with a crude Gaussian-shaped curve, the AROME model was able to simulate a more detailed diurnal cycle. However, the high-resolution model AROME also underestimated the TKE daytime maxima at the lowest model level, which is in agreement with the present findings. In our case, the turb_hybrid scheme led to a successful simulation of TKE in the afternoon, because the up-valley flow can be associated with strong horizontal shear production (Weigel et al. 2006).

For many NWP models the representation of the night-time stable ABL is even over flat terrain a challenge, since there are various phenomena interacting at different scales (Mahrt 2014). In mountainous terrain, katabatic flows, drainage flows, meandering motions, density currents, and intermittent turbulence add more complexity to the problem (Schmidli et al. 2009; Zhou and Chow 2013). While some of these phenomena may be well resolved at a horizontal grid spacing of $\Delta x=1.1\, \hbox {km}$, the model horizontal and vertical grid spacing is still too coarse for the simulation of many small-scale processes in the stable ABL, such as very shallow katabatic flows. Therefore, the model shows mixed results in adequately simulating the night-time TKE, especially at the slope station with the turb_1D scheme, where the simulated TKE values decrease to zero. With the turb_hybrid scheme, the model is able to simulate non-zero TKE during the night, which is mainly due to the vertical turbulent transport. Since the buoyancy production, which is underestimated during night-time, is related to the sensible heat flux and the longwave radiation, a closer investigation of the model radiation and energy balance is necessary.

It is often discussed at which horizontal grid spacing the introduction of a 3D turbulence scheme is advisable (Honnert and Masson 2014). Introducing a horizontal length scale in our complex terrain setting with $\Delta x=1.1\, \hbox {km}$ is clearly beneficial: terrain-related 3D effects, especially horizontal shear contributions, lead to a better model performance with the turb_hybrid scheme (Eq. 5). Furthermore, the verification also suggests improvement in TKE on the slopes, thus indicating that the turb_hybrid scheme is also more suitable for inclined surfaces. This is supported by the vertical profiles at the steep north-facing slope (Fig. 10), where the turb_hybrid scheme leads to the simulation of higher TKE values throughout the ABL.

Despite the apparently positive impact of introducing horizontal shear production, it may be questioned whether indeed shear processes are significant at the scales under consideration (i.e., 1.1 km for the present simulations) to produce turbulence. From a classical boundary-layer point of view, horizontal shear production is expected to occur on scales of the order of a few hundred metres (and hence entirely subgrid). The topographic setting in the present environment, however, indeed introduces a systematic horizontal shear mostly perpendicular to the valley axis and related to the core of the up-valley flow (Fig. 3b), which at least has the potential to generate sufficiently strong shear over scales $\sim 1 \hbox { km}$. Furthermore, the interaction of the along-valley flow and the cross-valley slope circulation can trigger turbulence-like perturbations at the scales of interest (Wagner et al. 2015b). As often, however, when physical parametrizations interfere with resolution (see, e.g., the convection problem) the scales cannot be completely separated. Here, the meso-scale flow (which is resolved at $\Delta x=1.1\, \hbox {km}$) triggers turbulence at scales that are not entirely subgrid—but also not completely resolved, which is a manifestation of the grey zone described by Wyngaard (2004). To what degree the chosen parametrization of horizontal shear production adequately (qualitatively and quantitatively) represents the physical processes at work requires further investigation.

The use of the horizontal grid spacing in LES as a length scale might not be suitable for other applications since it was initially developed to keep the overall circulation stable in flows with large horizontal gradients (Smagorinsky 1990). We also conducted test simulations on a horizontal grid spacing of $\Delta x=2.2\, \hbox {km}$ for 1 July 2015. While horizontal grid spacing has no significant impact on the TKE production when using the turb_1D scheme, TKE is largely overestimated in the turb_hybrid scheme (not shown), reaching values of up to $7\, \hbox {m}^{2} \, \hbox {s}^{-2}$ during the up-valley flow phase, which is clearly unrealistic. Therefore, a length scale on the order of 1 km, which appears to be a “lucky choice“ in the present simulations with $\Delta x=1.1\, \hbox {km}$, seems to be appropriate, at least for the present topography and possibly the chosen weather situations. Conversely, the horizontal grid spacing as a substitute for the turbulence length scale in the parametrization of horizontal shear production of TKE is not generally an appropriate choice. Since the grid length as a horizontal length scale is not based on a physical background at our chosen horizontal grid spacing, further investigation is required to determine a horizontal length scale that is also physically plausible (e.g. dependent on the current ABL structure).

## Summary and Conclusions

We performed numerical simulations with the state-of-the-art NWP model COSMO in complex terrain. Modelled TKE is compared with TKE observations at the so-called i-Box stations, where each flux tower is located at a representative surface in complex terrain. More importantly, we have not only validated the model on the basis of the mean flow characteristics (e.g., how well the model simulates the onset, strength and duration of the daytime up-valley flow and the associated temperature structure), but also the performance of the turbulence closure: the evolution of TKE is evaluated and compared to measurements at various sites. Moreover, and probably for the first time (at least for complex terrain, and very high resolution), not only TKE, but also the various terms in the TKE budget equation are evaluated against measurements.

The results show that the model 1D turbulence parametrization is not able to simulate the TKE evolution correctly for typical atmospheric processes in complex terrain, e.g. during the daytime up-valley flow or downslope flows during the night-time. Introducing 3D effects (horizontal shear production, advection of mean TKE, so-called hybrid turbulence parametrization) into the TKE budget equation has a significant impact: the horizontal contributions to shear yield a more realistic TKE structure in the valley. This leads to the following major conclusions:

1. The general improvement in TKE simulation with the hybrid turbulence parametrization suggests that typical 1D turbulence parametrizations are not suitable for a complex terrain environment and the associated atmospheric boundary-layer processes.
2. In situations when buoyancy-driven TKE is dominant, mostly before noon, we found a generally realistic model performance even with the 1D turbulence parametrization and only a slightly better simulation of TKE with the hybrid turbulence parametrization.
3. For shear-dominated situations with a strong up-valley flow, horizontal shear production significantly contributes to the TKE budget, leading to a realistic simulation of the TKE with shear as the main source term.
4. During the night-time when a stable boundary layer forms, vertical turbulent transport is a local source term for TKE. With the hybrid turbulence parametrization, we found a major improvement of simulated TKE on the steep slope, mostly related to better-simulated vertical turbulent transport.
5. The improvement at the slope stations (also during night-time) suggests that 3D effects are crucial for the TKE structure on sloped surfaces. Hence, a better representation of slope flows also improves the simulation of thermally-driven circulations and exchange processes with the free troposphere.
6. Mean advection of TKE has a minor influence on the near-surface TKE structure for our investigated weather situations. However, this might change for strong foehn winds, which occur frequently in and around the city of Innsbruck and surroundings.
7. The hybrid turbulence parametrization uses the grid spacing as a turbulence length scale in the parametrization of horizontal shear production. Clearly, the good results emphasize the importance of horizontal shear for TKE simulation in complex topography. While the horizontal grid spacing employed in the present study ($\Delta x=1.1\, \hbox {km}$) was obviously favourable, the question remains how to more generally define a turbulence length scale suitable for the description of horizontal shear production of TKE. For this, simulations on a finer horizontal grid spacing will be instrumental.

It is finally noted that the improved TKE parametrization has only relatively minor implications on the mean flow structure in the valley, so that a traditional verification would possibly not have revealed the present results. The importance of improved TKE representation lies within the model (e.g., in a potentially more reliable fog parametrization, snow-cover simulation, or shallow convection), but also, and maybe more importantly, as an input for offline simulations. If a high-resolution NWP model is used to provide input for a follow-up simulation—such as assessing the wind-energy potential at hub height, hydrological simulations, pollen forecasting, or pollutant dispersion—it is most often the turbulence state, as represented by TKE, that needs to be well-simulated, and not only the mean meteorological fields.

## Footnotes

1 http://www.ecmwf.int/en/forecasts/datasets/set-i.

2 http://www.jpl.nasa.gov/news/news.php?release=2009-103.

3 http://webarchive.iiasa.ac.at/Research/LUC/External-World-soil-database/HTML/.
